# Cancer Organoids in Basic Science and Translational Medicine

**DOI:** 10.3390/cancers13153701

**Published:** 2021-07-23

**Authors:** Lorenzo Memeo, Vincenzo Canzonieri, Flavio Rizzolio

**Affiliations:** 1 Department of Experimental Oncology, Mediterranean Institute of Oncology, 95029 Catania, Italy; 2 Department of Pathology, IRCCS CRO Aviano National Cancer Institute, 33081 Aviano, Italy; vcanzonieri@cro.it (V.C.); flavio.rizzolio@unive.it (F.R.); 3 Department of Medical Surgical and Health Sciences, University of Trieste, 34127 Trieste, Italy; 4 Department of Molecular Science and Nanosystems, Università Ca’ Foscari Venezia, 30170 Venice, Italy

Organoids are revolutionizing approaches to cancer therapy and even diagnosis. Organoids are the 3D culture of self-organized cells with stem cell properties that differentiate in specific cellular functions depending on their tissue of origin. Cancer organoids could recapitulate the architecture, functions, genetic, epigenetics, and the pathophysiological hallmarks of cancer. This Special Issue covers different aspects of cancer organoids and their applications in the oncology field.

The research paper from Papazoglou et al. [1] evaluated the effects of Cisplatin and Pemetrexed on cell viability using a homologous cell-derived extracellular matrix (hECM) as substratum and subsequently in the following 3D cell culture phenotypes: tumor spheroid formation, tumor spheroid invasion, and collagen gel contraction, using benign mesothelial cells as a control. Their results showed that an in vitro 3D platform can be used for testing novel drugs against malignant mesothelioma to improve the effects of first-line chemotherapeutics in this highly aggressive tumor.

Sensi et al. [2] proposed a patient-derived 3D preclinical model for drug evaluation in Stage II and III colorectal cancer using recellularized scaffolds and performing a chemosensitivity test for 5-FU. In this way, the authors demonstrated that the drug diffused through the repopulated 3D CRC scaffolds and co-localized within the cell nuclei. Their data confirmed that the bioengineered CRC 3D model could be a reliable preclinical patient-specific platform to bridge the gap between in vitro and in vivo drug testing assays and provide effective cancer treatment.

Hum et al. [3] compared the transcriptional profile of 4T1 murine mammary carcinoma cells from 2D and 3D cultures, subcutaneous or orthotopic allografts, as well as ex vivo tumoroids. The 3D culture platforms had more in vivo-like transcriptional profiles than 2D cultures. In vivo tumors had more cells undergoing epithelial-to-mesenchymal transition (EMT) while in vitro cultures had cells residing primarily in an epithelial or mesenchymal state. Ex vivo tumoroids incorporated aspects of in vivo and in vitro culturing, retaining a higher abundance of cells undergoing EMT while shifting cancer cell fate towards a more mesenchymal state. Cellular heterogeneity surveyed by scRNA-seq revealed that ex vivo tumoroids, while rapidly expanding cancer and fibroblast populations, lost a significant proportion of immune components. These data emphasize the need to improve in vitro culture systems and preserve the syngeneic-like tumor composition by maintaining similar EMT heterogeneity as well as the inclusion of stromal subpopulations.

Sondorp et al. [4] developed a cancer organoid model with the potential for pre-treatment diagnosis of patients with papillary thyroid carcinoma (PTC) that is I^131^-resistant. The authors compared organoids obtained from thirteen patients with PTC with organoids from I^131^-resistant PTC from three patients, revealing a substantial difference at both protein and gene expression levels, indicating treatment prediction potential.

Campaner et al. [5] established patient-derived tumor organoids (PDTOs) from different breast cancer subtypes, which showed histological and genomic concordance with parental tumors, demonstrating that organoids represent a valuable system to test the efficacy of standard therapeutic treatments and to identify drug-resistant populations within tumors.

The review from Elbadawy et al. [6] described various prostate cancer models with a special focus on organoids and their values in basic medicine, personalized therapy, and translational research in vitro and in vivo, which could help to achieve the full transformative power of cancer precision medicine.

Lin et al. [7] provided an interesting review summarizing how the development of in vitro cancer organoids in recent years has demonstrated potential to improve therapies for patients with pancreatic duct adenocarcinoma.

Similarly, Frappart et al. [8] reviewed the current technical and scientific knowledge on pancreatic duct adenocarcinoma organoids, their future perspectives, and how they can represent a pivotal change in the fight against pancreatic duct adenocarcinoma by improving both diagnosis and treatment options.

Another interesting review by Gilazieva et al. [9] explored the use of three-dimensional (3D) tumor models such as spheroids and organoids using high-throughput personalized medicine methods to provide a suitable therapy for patients. Advantages and disadvantages are highlighted in the text, suggesting possible directions to improve (3D) tumor models for a better personalized therapy.

Andreatta et al. [10] summarized in a review how engineered brain organoids via gene editing and co-cultures of patient-derived tumor spheroids with stroma cells have enabled the analysis of cancer development in a context that better mimics brain tissue architecture. Moreover, the establishment of biobanks from glioblastoma patient-derived organoids represents a crucial starting point to improve targeted therapies.

Vivarelli et al. [11] described in detail the role of PDTOs as a platform to validate the efficacy of anti-cancer drugs, how PDTO libraries are helping the discovery of novel anticancer molecules, and how PDTOs represent a good model to screen and validate compounds employed for other pathologies as off-label drugs potentially repurposed for the treatment of tumors. An interesting analysis of current clinical trials focused on organoids was provided.

Finally, our group described [12] how microfluidic organoid or organ-on-a-chip platforms are a new group of promising microengineered models that recapitulate 3D tissue structure and physiology and combine several advantages of current in vivo and in vitro models.

In this Special Issue, an overview is provided of the application of organoid technology in different cancer types including breast, colorectum, pancreatic, thyroid, and mesothelioma. A combination of research articles and reviews highlights the advantages and disadvantages of this technology, with the overall conclusion that organoids are the most advanced in vitro and ex vivo technologies, with high physiological relevance for understanding the functional pathways involved in tumor evolution and to improve the diagnosis and therapy of cancer patients. Clinical trials are ongoing to assess the real impact of organoids in clinics.
